# Bone marrow mesenchymal stem cells protect against cerebral amyloid angiopathy by enhancing neutrophil mitocytosis

**DOI:** 10.4103/NRR.NRR-D-24-01273

**Published:** 2025-06-19

**Authors:** Mengyan Hu, Haotong Yi, Shisi Wang, Xinmei Kang, Yuxin Liu, Zhiruo Liu, Huipeng Huang, Qin Qin, Liling Yuan, Wei Cai, Wei Qiu, Zhengqi Lu, Sanxin Liu

**Affiliations:** 1 Department of Neurology, Mental and Neurological Disease Research Center, the Third Affiliated Hospital of Sun Yat-sen University, Guangzhou, Guangdong Province, China; 2 Guangdong Provincial Key Laboratory of Brain Function and Disease, Guangzhou, Guangdong Province, China

**Keywords:** bone marrow mesenchymal stem cell, cerebral amyloid angiopathy, cognitive decline, migrasome, mitochondria, neutrophil mitocytosis

## Abstract

Current treatments for cerebral amyloid angiopathy are mainly symptomatic and have limited efficacy, and there is a lack of targeted therapies. Mesenchymal stem cell transplantation improves cognitive and motor function in conditions such as Alzheimer’s disease, acute ischemic stroke, and Parkinson’s disease. In addition, mesenchymal stem cell therapy modulates the immune system, reduces neuroinflammation, and improves resolution of brain lesions by cells of the macrophage lineage. Cerebral amyloid angiopathy and Alzheimer’s disease share similar pathologic changes involving amyloid-beta deposition, which contributes to the progression of both diseases and exacerbates cognitive deficits through impaired vascular integrity and neuroinflammation. Therefore, we hypothesized that mesenchymal stem cell therapy could also ameliorate the pathological changes seen in cerebral amyloid angiopathy by modulating the immune response. In this study, we show that bone marrow mesenchymal stem cells have a protective effect in a mouse model of cerebral amyloid angiopathy (Tg-SwDI/B). Bone marrow mesenchymal stem cell treatment improved cognitive function, reduced neuroinflammation, and maintained blood–brain barrier integrity in Tg-SwDI/B mice. Mechanistically, bone marrow mesenchymal stem cell treatment enhanced the expulsion of damaged mitochondria from neutrophils via migrasomes, in a process known as mitocytosis, thereby preserving mitochondrial quality within the neutrophils. Mitochondrial damage in neutrophils leads to cellular injury, including the generation of reactive oxygen species and the formation of neutrophil extracellular traps. Neutrophils activate mitocytosis to promote mitochondrial renewal, which further enhances their own clearance by macrophage lineage cells. Our findings demonstrate that bone marrow mesenchymal stem cells are a promising therapeutic candidate for cerebral amyloid angiopathy, as they play a significant role in migrasome-dependent mitochondrial quality control in neutrophils.

## Graphical abstract

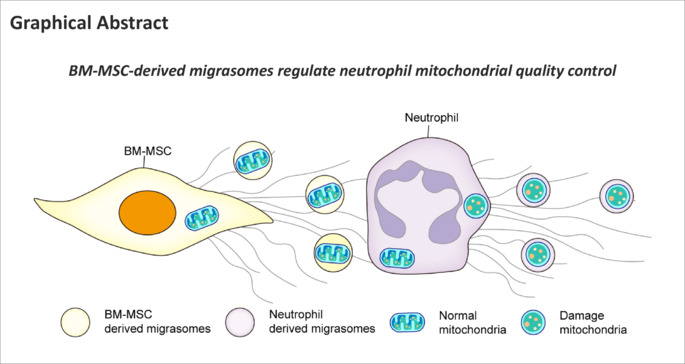

## Introduction

Cerebral amyloid angiopathy (CAA) is the most common age-related small vessel disease of the brain (Boyle et al., 2015; Jäkel et al., 2022). Current therapies for CAA are largely symptomatic and have limited efficacy with regards to addressing the underlying disease mechanisms (Cozza et al., 2023; Wang et al., 2024). Conventional treatments do not target the specific mechanisms of CAA, and thus fail to slow disease progression or improve patient outcomes (Cozza et al., 2023). Therefore, there is an urgent need for novel therapeutic approaches that directly address the pathophysiological processes of CAA and offer more effective treatment options.

Multiple studies have shown that adoptive transfer of mesenchymal stem cells (MSCs) improves cognitive and motor functions in patients with conditions such as Alzheimer’s disease (AD) (Qin et al., 2022), acute ischemic stroke (AIS) (Tian et al., 2023), and Parkinson’s disease (Andrzejewska et al., 2021; Zhuo et al., 2024). Additionally, research indicates that MSCs effectively mitigate neuroinflammation, improve cerebral blood flow, and bolster cognitive function in AD (Bonsack et al., 2020; Hernández and García, 2021). MSC therapy has been shown to induce significant improvements in neurological function, increase neurogenesis, and reduce infarct volume in the ischemic brain in animal studies (Kim et al., 2020; Li et al., 2023a). Furthermore, MSC therapy has been shown to modulate the immune system, reduce neuroinflammation, and improve resolution of brain lesions by macrophage lineage cells (Ceccariglia et al., 2019). CAA and AD share similar pathological changes involving amyloid-beta (Aβ) deposition, which contributes to the development of both conditions and exacerbates cognitive impairment through impaired vascular integrity and neuroinflammation (Greenberg et al., 2020). Therefore, we hypothesized that MSC therapy may also improve the pathological changes seen in CAA by regulating immune responses.

MSCs exert their therapeutic effects through various mechanisms, including the release of extracellular vesicles (EVs) and soluble factors (Giovannelli et al., 2023). Previous studies have demonstrated that mitochondrial transfer through tunneling nanotubes is a crucial mechanism by which MSCs exert their protective effects (Jackson et al., 2016; Baldwin et al., 2024). Additionally, we previously showed that bone marrow MSCs (BM-MSCs) release migrasomes, organelles that form during cellular migration (Li et al., 2023a). Migrasomes play a role in maintaining other intracellular organelles, such as mitochondria (Jiang et al., 2023). Damaged mitochondria are removed from the cell via migrasomes in a process called mitocytosis, to support the mitochondrial membrane potential and respiration (Jiao et al., 2021). BM-MSCs have been reported to transfer mitochondria to other cells, and we hypothesize that this transfer may occur via migrasomes (Malekpour et al., 2023). However, neutrophil mitocytosis has been observed in response to various stimuli and plays a role in mitochondrial quality control in these cells (Zhao et al., 2021). Mitochondrial structural damage and dysfunction, characterized by increased oxidative damage, impaired respiration, and disrupted substrate utilization, are well-documented (Sorrentino et al., 2018). It is crucial for neutrophils to preserve mitochondrial health, which helps avoid local activation at lesion sites, and to migrate to the bone marrow or other central immune organs for clearance (Adrover et al., 2019; Fairley et al., 2022). Whether neutrophil mitocytosis contributes to Aβ deposition and cognitive decline in CAA remains to be elucidated.

BM-MSCs have multiple regulatory effects on the immune system (Wang et al., 2022). We previously demonstrated that BM-MSCs enhance macrophage phagocytic function (Li et al., 2023a). In CAA, macrophages are critical for clearing Aβ and removing proinflammatory immune cells (Hu et al., 2023; Uekawa et al., 2023). However, it remains unclear whether BM-MSCs also improve macrophage-mediated clearance of Aβ in CAA. Phagocytic activity in macrophages is governed by complex mechanisms. Additionally, EVs derived from neutrophils significantly enhance macrophage phagocytic efficiency, while macrophages are involved in clearing neutrophils at the lesion site (Nepal et al., 2019; Uderhardt et al., 2019). The intricate interactions among immune cells influence CAA progression. Further research is necessary to elucidate how BM-MSCs mediate interactions among the various immune cells present within CAA lesions.

In this study, we explored the protective effects of MSC against CAA progression. We report that adoptive transfer of BM-MSCs protects against CAA. BM-MSC treatment improved cognitive function, reduced neuroinflammation, and maintained blood–brain barrier (BBB) integrity in Tg-SwDI/B mice. Mechanistically, the BM-MSCs transferred their own healthy mitochondria to neutrophils, which facilitated the exocytosis of damaged mitochondria from neutrophils, thereby preventing intracellular oxidative stress. Oxidative stress, often resulting from mitochondrial dysfunction, is a key factor in the formation of detrimental neutrophil extracellular traps (NETs) and the excessive production of pro-inflammatory mediators by neutrophils (Papayannopoulos, 2018). By renewing their mitochondria, neutrophils are better equipped to avoid these harmful effects, ensuring their proper withdrawal or clearance by other phagocytes (Cai et al., 2020). In the brain, macrophages play a central role in scavenging neutrophils (Cai et al., 2020; Zhao et al., 2024). Additionally, neutrophil-derived migrasomes enhance their clearance by phagocytes (Cai et al., 2020). Our finding suggests that BM-MSCs orchestrate intricate immune cell interactions in CAA and represent a promising therapeutic strategy for this condition.

## Methods

### Animals

A total of 50 male wild-type C57BL/6J (WT) mice (8 months old; body weight: 30–50 g; GemPharmatech, Nanjing, China; License No. SCXK(Yue)2020-0054) and 40 male homozygous Swedish-Dutch-Iowa transgenic (Tg-SwDI/B) mice (8 months old; C57BL/6J background; body weight: 30–50 g; The Jackson Laboratory, Bar Harbor, ME, USA, MSR Cat# JAX: 007027) were used. Additionally, 20 male WT C57BL/6J mice (8–12 weeks old; body weight: 20–30 g; GemPharmatech; Cat# N000013) were included for primary bone marrow-derived neutrophil and macrophage cultures. All aged WT and Tg-SwDI/B mice were subjected to behavioral tests, followed by post-mortem analyses including immunofluorescence staining, senescence-associated beta galactosidase (SA-β-Gal) staining, BBB integrity assessment, transmission electron microscopy, quantitative determination of mRNA expression, and flow cytometric analysis of immune cell populations. Tg-SwDI/B mice were randomly assigned to two groups (*n* = 20/group): CAA model group: intravenous injection of vehicle (PBS) at matched time points; MSC-CAA group: intravenous administration of BM-MSCs (2 × 10^6^ cells in phosphate buffered saline (PBS) per mouse, injected via the inner canthus vein, in two doses of 200 μL, with a 3-day interval) starting at 8 months of age. Tg-SwDI/B mice were generated by knocking in the human Aβ precursor protein gene carrying three familial mutations known to be associated with CAA (Davis et al., 2004). The mice used for all experiments were naïve and had not undergone any drug testing. All mice were housed in a specific pathogen-free facility under a 12/12-hour light/dark cycle, with the temperature maintained at 24 ± 2°C and the humidity between 30% and 70%. They had *ad libitum* access to food and water and were housed five per cage. Healthy WT C57/BL6 mice were used as controls for the Tg-SwDI/B mice. Behavioral tests were conducted starting on day 7 after BM-MSC injection, and the mice were euthanized on day 11 under anesthesia induced by isoflurane (5% at 1 L/min for induction and 1% at 1 L/min for maintenance, in a mixture of 30% O_2_ and 70% N_2_O) (RWD, Shenzhen, China, Cat# R510-22). The Animal Care and Ethics Committee at the Institute of Biological and Medical Engineering, Guangdong Academy of Sciences granted approval for the animal studies (approval No. K2024-01-100-485) on July 9, 2024. All animal experiments were conducted in accordance with the National Institutes of Health Guide for the Care and Use of Laboratory Animals (8^th^ ed., National Research Council, 2011). All experiments were designed and reported according to the Animal Research: Reporting of *In Vivo* Experiments (ARRIVE) guidelines (Percie du Sert et al., 2020).

### Isolation of human bone marrow mesenchymal stem cells

Heparin-treated bone marrow was obtained from healthy donors (recruited through advertisement at the Third Affiliated Hospital of Sun Yat-sen University) who provided informed consent. Bone marrow mesenchymal stem cells (BM-MSCs) were derived from the bone marrow using Ficoll-Paque (with a density of 1.077 g/mL, Cat# 17-1440-02 from GE (Healthcare (Hyclone), Chicago, IL, USA) and density gradient centrifugation. The cells were then cultivated in MSC SFM basal medium (Gibco, Waltham, MA, USA, Cat# A13829-01) enriched with MSC SFM supplement (Gibco, Cat# A11577-01). The BM-MSCs were pre-treated with blebbistatin (BLEB, MCE, Shanghai, China, Cat# HY-13441, 50 μM for 24 hours) to inhibit migrasome formation and then treated with Aβ_40_ (MCE, Shanghai, China, Cat# HY-P0265, 20 μg/mL for 2 hours). The Ethics Committee of the Stem Cell Clinical Research of the Third Affiliated Hospital of Sun Yat-sen University approved this study (approval No. 2022-2) on September 2, 2022. All experiments involving BM-MSCs were conducted in accordance with the principles outlined in the 2021 ISSCR Guidelines for Stem Cell Research and Clinical Translation.

### Behavioral tests

Tg-SwDI/B mice at 8 months of age and age- and sex-matched WT mice were used for behavioral tests. All behavioral tests were recorded and analyzed by investigators who were blinded to the group assignments.

#### Novel object recognition

The novel object recognition test was used to assess object recognition memory in the mice. One hour before the test, each mouse was allowed to explore two identical objects. One of the training objects was then replaced with a novel object that differed in shape and color but was similar in height and volume. Mice naturally prefer novelty, so they tend to spend more time exploring new objects than familiar ones. Motor activity and time spent exploring the familiar (F) and novel (N) objects were recorded using EthoVision XT (Noldus Information Technology, Wageningen, the Netherlands). Both the recognition index (RI, N/(N + F)) and the discrimination index (DI, (N−F)/(N + F)) were calculated to evaluate mouse performance.

#### Morris water maze test

Cognitive function in mice was assessed using the Morris water maze test. During the learning phase, a square platform (11 cm × 11 cm) was submerged 2 cm beneath the water surface in a circular pool (diameter 150 cm) filled with opaque water. The mice underwent four serial probe tests (one test per day, starting from 7 days after BM-MSC transfer) and a clued test (11 days after BM-MSC transfer) of the Morris water maze. In the probe tests, the mice were placed in the pool at one of four starting locations and given 60 seconds to locate the hidden platform. Each mouse underwent four trials per day, with randomly assigned starting positions, over four consecutive days of training. At the end of each trial, the mouse was placed on the platform and allowed to remain there for 30 seconds, during which prominent spatial cues were displayed around the room. The time taken to reach the platform was recorded. In the clued test performed on day 5, the platform was removed, and a single 60-second probe trial was performed. The time spent in the goal quadrant where the platform had previously been located was recorded.

### Culturing primary mouse bone marrow–derived neutrophils

To isolate neutrophils from the bone marrow, we extracted bone marrow from the femurs and tibias of 20 healthy male WT mice, aged 8–12 weeks. The marrow cells were then suspended in Hank’s Balanced Salt Solution (HBSS; Gibco) and subjected to centrifugation in a 58.5%/65% Percoll gradient at 18°C at 800 × *g* for 30 minutes. The neutrophils were retrieved from the interface layer, rinsed with HBSS (Gibco), and cultured in Roswell Park Memorial Institute (RPMI) 1640 medium (Gibco, Cat# 1640-c11875500bt) supplemented with 10% fetal bovine serum (FBS; Gibco) and 1% penicillin-streptomycin (PS; Gibco) for subsequent *in vitro* experimentation. Bone marrow–derived neutrophils were isolated from WT mice and co-cultured with BM-MSCs at a ratio of 5:1 for 2 hours.

### Isolation of neutrophil/mesenchymal stem cell-derived migrasomes

Neutrophils and MSCs were grown in complete medium (Nuwacell, Hefei, China, Cat# RP02010) in 150-mm petri dishes coated with a 0.1 μg/mL fibronectin solution for 12 hours. The cells and migrasomes in the dishes were exposed to a 0.25% trypsin solution and then transferred to 50-mL conical tubes. All subsequent steps were carried out at 4°C. The samples were centrifuged to remove cells and larger debris, starting with centrifugation at 1000 × *g* for 10 minutes, followed by a more intense centrifugation at 4000 × *g* for 20 minutes. The migrasomes were then pelleted by centrifugation at 20,000 × *g* for 30 minutes. The raw migrasome pellets were washed with PBS and further centrifuged twice at 20,000 × *g* for 30 minutes before proceeding to additional analysis or *in vitro* use.

### Culturing primary mouse bone marrow–derived macrophages

Macrophages derived from primary bone marrow were obtained by extracting cells from the femurs and tibias of healthy WT C57BL/6 mice aged between 8 and 12 weeks. The macrophage precursors were grown and induced to differentiate into mature macrophages over 6 days in RPMI 1640 medium enriched with MCSF, 10% FBS, and 1% penicillin-streptomycin (PS).

### Phagocytic function assay

Bone marrow–derived neutrophils were isolated from WT C57/BL6 mice at 8–12 weeks of age, treated with Aβ40 (20 μg/mL for 2 hours), and co-cultured with BM-MSCs (5:1). Neutrophil-derived migrasomes were isolated and applied to primary mouse bone marrow–derived macrophages (50 μg/mL for 24 hours). Neutrophils were incubated overnight in a PBS solution with 1% FBS to trigger apoptosis. To assess efferocytosis capacity, the apoptotic neutrophils were stained with the dead cell indicator propidium iodide (PI; Invitrogen, Carlsbad, CA, USA, Cat# P1304MP) at a concentration of 1 μg/mL for 15 minutes at 37°C. Subsequently, these stained neutrophils were co-cultured with macrophages at a ratio of five dead neutrophils to one macrophage for 1 hour. For the purpose of *in vitro* immunostaining, macrophages were initially cultured on coverslips that had been coated with poly-l-lysine. The coverslips were then rinsed twice to eliminate any non-engulfed neutrophils and fixed using 4% paraformaldehyde. After fixation, the coverslips underwent immunostaining, were meticulously removed using tweezers, and then placed onto microscope slides. The F-actin within the macrophages was labeled with Alexa Fluor 488 phalloidin (1:500, Invitrogen, Cat# A12379) at room temperature in a dark environment for 30 minutes.

### Organotypic brain slice culture

Organotypic brain slices were obtained from the cerebrum of 10 postnatal day 7 mouse pups (WT mice), sectioned sagittally at 500 μm intervals using a McIlwain tissue chopper (CAVEY LAB, MTC-21), and transferred to six-well culture inserts (pore diameter 0.4 μm). CO_2_ anesthesia was administered via inhalation, typically using a concentration of 5% to 10% CO_2_ (Airgas, Radnor, PA, USA). The slices were cultured in 1 mL of OSC medium, composed of 36.7% Basal Medium Eagle (BME; Thermo Fisher Scientific, Waltham, MA, USA), 36.7% Neurobasal-A Medium (Thermo Fisher Scientific), 1% GlutaMAX (Gibco), 0.033% insulin (Beyotime, Shanghai, China), 0.5% penicillin-streptomycin, and 25% heat-inactivated horse serum (GE Hyclone, Chicago, IL USA). After 24 hours, the OSC medium was replaced with fresh medium after 24 hours and then every 2 days until day 7.

### Blood–brain barrier integrity assessment

BBB integrity in mice was assessed by BBB permeability analysis. First, a single dose of 3 kDa-Dextran (3 kDa-Dex, labeled with Cy3, Ruixibio D-Y1002551) was administered intravenously to the mice (10 mg/kg) 90 minutes before sacrifice. After collecting peripheral blood, the mice were perfused with PBS (10 mL), followed by 4% paraformaldehyde (Biosharp, Cat# BL539A, 10 mL). The fluorescence intensity of the 3 kDa-Dextran in the plasma was measured using a 96-well plate reader (Biotek Synergy H1MF). The extravasation index of 3 kDa-Dextran in the brain parenchyma was calculated as the ratio of extravascular to intravascular 3 kDa-Dextran MFI (ImageJ, Version 1.54, National Institutes of Health, Bethesda, MD, USA) and further normalized to that of the WT group.

### Flow cytometric analysis

Flow cytometry was performed utilizing a FACS analyzer (BD Biosciences, San Jose, CA, USA). Mouse peripheral blood samples were collected with heparin as the anticoagulant, followed by the removal of red blood cells using ACK lysis buffer (Gibco, Cat# A1049201). The leukocytes were rinsed in PBS prior to surface marker labeling. The brain was processed into a single-cell suspension for flow cytometry. The ipsilateral hemispheres were extracted from the dissected brains, digested with 0.25% trypsin-EDTA at 37°C for 25 minutes, and subsequently strained through a 70-μm filter to obtain a single-cell suspension. Neuronal cells were purified from myelin debris via centrifugation through a 30%/70% Percoll gradient. The cells at the interface were retrieved, rinsed with PBS, fixed, permeabilized, and stained. The following antibodies were employed for surface marker identification: PerCP-labeled anti-mouse CD45 (1:400, Biolegend, San Diego, CA, USA, Cat# 103132, clone 30-F11, RRID: AB_893340), FITC-labeled anti-mouse CD3 (1:400, Biolegend, Cat# 100204, clone 17A2, RRID: AB_312661), PE-labeled anti-mouse CD19 (1:400, Biolegend, Cat# 152408, clone 1D3/CD19, RRID: AB_2629817), PE/CY7-labeled anti-mouse/human CD11b (1:400, Biolegend, Cat# 101216, clone M1/70, RRID: AB_312799), BV421-labeled anti-mouse F4/80 (1:400, Biolegend, Cat# 123132, clone 8M8, RRID: AB_11203717), FITC-labeled anti-mouse Ki67 (1:200, BD Pharmingen, San Jose, CA, USA, Cat# 556026, clone B56), and APC/CY7-labeled anti-mouse Ly6G (1:400, Biolegend, Cat# 108424, clone RB8-8C5, RRID: AB_2137485). After washing with PBS, the cells were fixed and permeabilized using Invitrogen’s Intracellular Fixation & Permeabilization Buffer Set (Carlsbad, CA, USA), followed by intracellular antibody staining. To assess the productivity of leukocyte-derived migrasomes, the cells were incubated with primary antibodies overnight. The primary antibody used was rabbit anti-TSPAN4 (1:200, Abcam, Cambridge, MA, USA, Cat# ab181995). The secondary antibody used was anti-rabbit Alexa Fluor 647 (1:1000, Abcam, Cat# ab150075, RRID: AB_2752244). A Live/Dead fixable stain kit (Invitrogen, Cat# L34957) was used to detect dead cells. Compensation for fluorochromes was achieved using single-stained OneComp eBeads (Invitrogen, Cat# 01-1111-41). Data analysis was performed with FlowJo software (version 10.0, FlowJo, Ashland, OR, USA).

### Senescence-associated beta galactosidase staining

SA-β-Gal staining was performed using an SA-β-Gal staining kit (Beyotime, C0602) according to the manufacturer’s instructions. Brain slices were fixed and stained with the staining solution overnight at 37°C in a non-CO2 incubator. Senescent cells were identified as blue-stained cells by light microscopy (Nikon Ti2-U).

### Immunofluorescence staining

Mice were euthanized at the designated time points. CO_2_ anesthesia is administered via inhalation, typically using a concentration of 5% to 10% CO_2_ (HOPE-MED8160, Tianjin, China). Following complete perfusion with 20 mL of PBS and 20 mL of 4% paraformaldehyde, the brains were extracted and slices into 25-μm coronal sections using a cryostat microtome (Leica, Wetzlar, Germany). The sections were then washed and incubated with primary antibodies at 4°C overnight in PBS containing 0.03% Triton X-100 and 3% BSA. After washing, the sections were incubated with secondary antibodies at room temperature for 1 hour. The primary antibodies used included: rabbit anti-neuron-specific nuclear protein (NeuN; 1:500, Abcam, Cat# ab177487), rabbit anti-MBP (1:500, Proteintech, Wuhan, China, Cat# 10458-1-AP), rabbit anti-c-FOS (1:500, Abcam, Cat# ab208942), rabbit anti-TSPAN4 (1:500, Abcam, Cat# ab181995), mouse anti-amyloid-β/6E10 (1:500, Biolegend, Cat# 803014), rat anti-CD31 (1:50, BD Pharmingen, Cat# 550274), rabbit anti-ZO1 (1:500, Abcam, Cat# ab221547), rabbit anti-CD45 (1:200, Abcam, Cat# ab281586), FITC-conjugated anti-mouse CD3 (1:500, Biolegend, Cat# 100204, clone 17A2), PE-conjugated anti-mouse CD19 (1:500, Biolegend, Cat# 152408, clone 1D3/CD19), rat anti-Ly6G (1:500, Proteintech, Cat# 65140-1-Ig), APC-conjugated anti-mouse F4/80 (1:500, Biolegend, Cat# 123116, clone BM8), and rabbit anti-Tom20 (1:200, Proteintech, Cat# 11802-1-AP). The secondary antibodies used were: anti-rat secondary antibody conjugated with Cy3 (1:1000, Jackson ImmunoResearch Laboratory, West Grove, PA, USA, Cat# 112-545-003), anti-rabbit secondary antibody conjugated with Cy3 (1:1000, Jackson ImmunoResearch Laboratories, Cat# 111-165-003), anti-rabbit secondary antibody conjugated with 488 (1:1000, Jackson ImmunoResearch Laboratories, Cat# 111-545-003), and anti-mouse secondary antibody conjugated with Alexa Fluor 488 (1:1000, Invitrogen, Cat# A-11059). Multiplex immunofluorescence staining was conducted using a PANO 7-plex kit (Panovue, Beijing, China) following the manufacturer’s instructions. DAPI Fluoromount-G (Abcam, Cat# ab104139) was used to visualize the nucleus when indicated. Images were captured with a confocal microscope (Leica TSC SP8) and processed with ImageJ software by a blinded observer who performed unbiased counting of automatically recognized cells and calculated the mean fluorescence intensity.

### Transmission electron microscopy

Fresh brain tissue samples (within 1 mm^3^ in volume) were swiftly harvested with a sharp-edged blade within 1–3 minutes of euthanasia. These tissue samples were placed into EP tubes filled with fresh transmission electron microscopy fixative for additional fixation and maintained at 4°C for safekeeping and transportation. Then, the brain tissues were washed three times with 0.1 M phosphate buffer (PB, pH 7.4, Servicebio, Wuhan, China) for 15 minutes each. Following fixation in the dark with 1% osmium tetroxide (OsO4, Ted Pella Inc, Redding, California, USA) in 0.1 M PB (pH 7.4) for 2 hours at room temperature, the tissues were rinsed three times with 0.1 M PB (pH 7.4) for 15 minutes each. Dehydration was performed at room temperature as follows: 30% ethanol (Sinopharm, Beijing, China) for 20 minutes; 50% ethanol for 20 minutes; 70% ethanol for 20 minutes; 80% ethanol for 20 minutes; 95% ethanol for 20 minutes; 100% ethanol for 20 minutes; and acetone (Sinopharm) for 15 minutes. Resin penetration and embedding were performed as follows: a 1:1 mixture of acetone and EMBed 812 (SPI, CA, USA) for 2–4 hours at 37°C; a 1:2 mixture of acetone and EMBed 812 overnight at 37°C; and pure EMBed 812 for 5–8 hours at 37°C. Pure EMBed 812 was then poured into embedding molds, and the tissue samples were placed in the resin. The molds were placed in a 37°C oven overnight. The embedding molds containing resin and samples were then transferred to a 65°C oven for polymerization for at least 48 hours. Next, the resin blocks were removed from the molds and stored at room temperature for subsequent use. Thin sections (60–80 nm) were cut from the resin blocks using an ultramicrotome and mounted onto 150-mesh copper grids coated with formvar film. The sections were stained in the dark with a 2% uranyl acetate solution in ethanol for 8 minutes, followed by three rinses in 70% ethanol and three rinses in ultrapure water. Subsequently, the sections were stained with 2.6% lead citrate in a CO_2_-free environment for 8 minutes and rinsed three times with ultrapure water. After drying on filter paper, the grids were placed on a grid board and left to dry overnight at room temperature. The tissue microstructure was examined with a HITACHI HT7800/HT7700 electron microscope (Hitachi, Tokyo, Japan).

### Mitocytosis analysis

In neutrophil or BM-MSC cultures, migrasomes were detected using wheat germ agglutinin (WGA, Invitrogen, Cat# W7024, 1 μg/mL, 15 minutes), which is a widely used lectin that binds to sialic acid and N-acetylglucosaminyl residues in the cell membrane. Mitochondria were stained with 150 nM Mito-tracker Red (Invitrogen, Cat# M7510) in serum-free media for 20 minutes at 37°C with 5% CO_2_, protected from light. After staining, the cells were washed three times with HBSS (Gibco). For co-staining with another antibody, fixation, permeabilization, blocking, and immunostaining were performed after Mito-tracker staining. The ratios of Mito-tracker^+^WGA^+^ migrasomes to WGA^+^ migrasomes were calculated. For *in vivo* neutrophil mitocytosis analysis, coronal brain sections were subjected to immunostaining for Ly6G, TSPAN4, and Tom20. The ratios of Ly6G^+^TSPAN4^+^Tom20^+^ migrasomes were calculated.

### Mitochondrial membrane potential assay

Mitochondrial membrane potential was assessed using a JC-1 mitochondrial membrane potential assay kit (Beyotime, Cat# C2003S). JC-1 dye accumulates in mitochondria in a potential-dependent manner, indicated by a fluorescence emission shift from green (**~**529 nm) to red (**~**590 nm). Mitochondrial depolarization is reflected by an increase in the green-to-red fluorescence intensity ratio. In brief, primary neutrophils were cultured and incubated with 5 μM JC-1 at 37°C for 30 minutes, followed by two washes with dilution buffer. JC-1 fluorescence was measured using a FACS flow cytometer (BD Biosciences). JC-1 monomers emit green fluorescence (**~**529 nm, detected with the FITC channel), while JC-1 aggregates emit red fluorescence (**~**590 nm, detected with the PE channel). The mitochondrial membrane potential was calculated as the ratio of green to red fluorescence.

### Quantitative determination of mRNA expression

Commercially available reagents from ESscience (Shanghai, China) were employed to isolate total RNA from cellular samples, following the protocol provided by the supplier. For first-strand cDNA production, a PrimeScript RT reagent kit (Takara, Shiga, Japan) was used in a 40-μL reaction mixture containing 1 μg of RNA with 260 nm/280 nm OD ratio between 1.8 and 2.2. Quantitative RT-PCR (qRT-PCR) was carried out using a QuantStudio 5 platform (ABI, Foster City, CA, USA), utilizing the TB Green Premix Ex Taq kit (Takara). Each reaction included 1 μL of cDNA and ROX. The qRT-PCR program was as follows: initial denaturation at 95°C for 30 seconds, then 40 cycles of denaturation at 95°C for 5 seconds and annealing/extension at 60°C for 34 seconds, and finally a melt curve analysis with stages at 95°C for 15 seconds, 60°C for 60 seconds, and 95°C for 15 seconds. The primer sequences used in this research are detailed in **Additional Table 1**. Data analysis was conducted using the comparative CT (2^–ΔΔCT^) method. Glyceraldehyde-3-phosphate dehydrogenase (GAPDH) served as the endogenous control for data normalization.

**Additional Table 1:** The primers used for quantitative reverse transcription-polymerase chain reaction

Gene	Forward primer (5’-3’)	Reverse primer (5’-3’)
**Mus musculus**		
*Tnf*	AGAAGTTCCCAAATGGCCTC	CCACTTGGTGGTTTGCTACG
*Ifna*	ACTTTGGATTCCCCCAGGAG	CTTAGGACAGGGATGGCTTTCT
*Ifng*	GCCACGGCACAGTCATTGA	TGCTGATGGCCTGATTGTCTT
*Il1a*	AAGACAAGCCTGTGTTGCTGAAGG	TCCCAGAAGAAAATGAGGTCGGTC
*Il6*	TCCTACCCCAACTTCCAATGCTC	TTGGATGGTCTTGGTCCTTAGCC
*Il10*	CCAAGCCTTATCGGAAATGA	TTTTCACAGGGGAGAAATCG
*1117*	CCTGGACTCTCCACCGCAA	TTCCCTCCGCATTGACACAG
*Il18*	GCCGACTTCACTGTACAACC	GTCTGGTCTGGGGTTCACTG
*Il16*	TTAGTCCAATCAGGGCGTGG	CACTGCATGAGTCCCTGACC
*Arg1*	CTCCAAGCCAAAGTCCTTAGAG	GGAGCTGTCATTAGGGACATCA
*Cell*	AGTTCTTGGCTCCACCAGAC	CATCCTGTATCCACACGGCA
*Cxcr1*	GGGTGAAGCCACAACAGATT	CGGTGTGTCAAAACCTCCTT
*Cxcr2*	CTTTGCCCTGACCTTGCCT	GCACAGGGTTGAGCCAAAA
*Mmp7*	CTGCCACTGTCCCAGGAAG	GGGAGAGTTTTCCAGTCATGG
*Mmp8*	CCACACACAGCTTGCCAATG	GCTTCTCTGCAACCATCGTG
*Mmp9*	CCAGCCGACTTTTGTGGTCT	TGGCCTTTAGTGTCTGGCTG
*C3*	ACCCCTTCATTCCTTCCACC	GAGTAATGATGGAATACATGGGGA
*Lta*	GCCTTTCCTGCCTTCGACTG	GTCATGTGGAGAACCTGCTGTG
*Gadph*	CCCTTAAGAGGGATGCTGCC	TACGGCCAAATCCGTTCACA

### Statistical analysis

Before proceeding with parametric statistical evaluations using Student’s *t*-test and one-way analysis of variance, normality was assessed. GraphPad Prism software (version 8.0, GraphPad Software, San Diego, CA, USA) was used for the statistical computations. When analyzing differences between two groups, the unpaired two-tailed *t*-test was applied. The error bars depicted in the figures represent the standard error of the mean (SEM). In cases where three or more groups were being compared, one-way analysis of variance was conducted without any matching or pairing, followed by Dunnett’s test to adjust the results for multiple comparisons. For the non-parametric tests in the Morris water maze experiment, the Wilcoxon rank-sum test was used, with the error bars indicating the SEM, as before.

## Results

### Adoptive transfer of bone marrow mesenchymal stem cells protects against cerebral amyloid angiopathy

**Figure 1:**
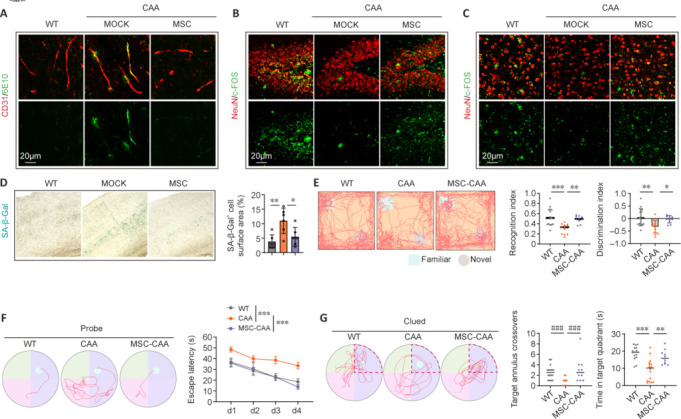
Adoptive transfer of BM-MSCs attenuates amyloid protein accumulation and improves cognitive function in a CAA model. Tg-SwDI/B mice with C57/BL6 background received BM-MSCs (2 × 10^6^ cells per mouse, i.v., in two doses with a 3-day interval) starting at 8 months of age. Behavioral tests were conducted starting on day 7, and the mice were euthanized on day 11. Age- and sex-matched WT mice served as controls. (A) Coronal brain sections from the recipients were collected and subjected to immunostaining for CD31 (red) and amyloid-beta (Aβ, clone: 6E10, green) to evaluate Aβ shedding in brain vessels. *n* = 6 in each group. (B, C) Neuronal activity in the hippocampus (B) and cortex (C) was evaluated by c-FOS expression in neurons (NeuN^+^). *n* = 6 in each group. (D) Coronal brain sections of the recipients were subjected to SA-β-Gal labeling. *n* = 6 in each group. Representative images are shown. Scale bar: 2 mm. (E) Recipients were subjected to novel object recognition tests (trained and tested 11 days after BM-MSC transfer). Motor activity and time spent in active exploration of familiar and novel objects during the test were quantified. *n* = 16 in the WT and CAA groups; *n* = 10 in the MSC-CAA group. (F, G) Recipient mice underwent four serial probe tests (F, one test per day, starting from 7 days after BM-MSC transfer) and a clued test (G, 11 days after BM-MSC transfer) of the Morris water maze. *n* = 16 in the WT and CAA groups; *n* = 10 in the MSC-CAA group. *P < 0.05, **P < 0.01, ***P < 0.001; one-way one-way analysis of variance followed by Dunnett’s test for D–F. Wilcoxon rank-sum test for G. CAA: Cerebral amyloid angiopathy. BM-MSCs: bone marrow mesenchymal stem cells; WT: wild-type.

To explore the therapeutic effect of BM-MSCs on CAA, Tg-SwDI/B mice (a CAA model) were treated with BM-MSCs (2 × 10^6^ cells per mouse, intravenously [i.v.]) starting at 8 months of age, when amyloid protein deposition is notably increased, according to previous reports (Davis et al., 2004). Treatment with BM-MSCs alleviated amyloid protein deposition in the cerebral vessels (**Figure 1A**) and enhanced neuronal activity in the hippocampus (**Figure 1B**) and cortex (**Figure 1C**) in the CAA mice. In addition, BM-MSC transfer reduced the accumulation of senescence-associated β-galactosidase (SA-β-Gal) in the parenchyma of Tg-SwDI/B mice (**Figure 1D**). BM-MSC recipients also demonstrated improved performance in the novel object recognition test (**Figure 1E**) and Morris water maze (**Figure 1F** and **G**). These results indicate that BM-MSC transfer protects against CAA by reducing amyloid protein deposition and SA-β-Gal accumulation, as well as improving cognitive function.

### Bone marrow mesenchymal stem cell transfer protects blood–brain barrier integrity in cerebral amyloid angiopathy

**Figure 2:**
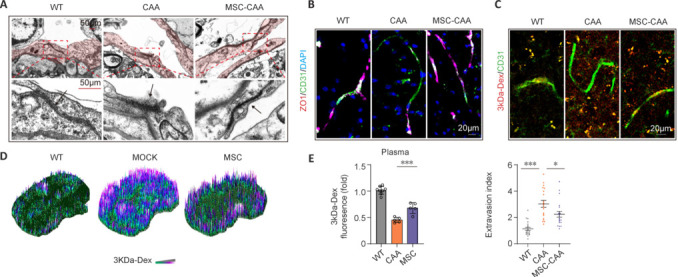
Transfer of BM-MSCs preserves the integrity of the blood–brain barrier in a CAA model. Tg-SwDI/B mice with C57/BL6 background received BM-MSCs (2 × 10^6^ cells per mouse, i.v., in two doses with a 3-day interval) starting at 8 months of age. Behavioral tests were conducted starting on day 7, and the mice were euthanized on day 11. Age- and sex-matched WT mice served as controls. (A) Representative images of transmission electron microscopy images of brain tissue from CAA and WT mice (age- and sex-matched). The arrows emphasize electron-dense regions representing tight junctions within the blood vessel wall (red tint). (B) Coronal brain sections from the recipients were collected and subjected to immunostaining for CD31 (green) and ZO-1 (red) to evaluate tight junction integrity. ZO1 coverage of brain vessels in the cortex was calculated. (C, D) Eleven days after the first treatment, the recipients were injected with 10 mg/kg 3 kDa dextran (Dex) 90 minutes before sacrifice. The mice were perfused with PBS and then 4% paraformaldehyde after peripheral blood collection. (C, D) Leakage of 3 kDa dextran in the brain parenchyma was assessed via fluorescence microscopy. *n* = 20 from three separate mice in each group. (E) The fluorescence intensity of 3 kDa dextran in the plasma of the recipients was assessed with a 96-well plate fluorescence reader. *n* = 8 in the WT group; *n* = 5 in the CAA and MSC-CAA groups. *P < 0.05, ***P < 0.001; one-way analysis of variance followed by Dunnett’s test. BM-MSCs: Bone marrow mesenchymal stem cells; CAA: cerebral amyloid angiopathy; DAPI: 4′,6-diamidino-2-phenylindole; WT: wild-type.

Disruption of the BBB is a key pathological feature of CAA and adversely affects prognosis (Hu et al., 2023; Inoue et al., 2023). We therefore investigated the protective effect of BM-MSCs on the BBB in Tg-SwDI/B mice. Our results demonstrated that BM-MSC treatment alleviated endothelial tight junction disruption, as evidenced by TEM (**Figure 2A**) and ZO-1 staining (**Figure 2B**). To evaluate BBB integrity, mice were injected with a single dose of 3 kDa-Dextran (3 kDa-Dex, 10 mg/kg, i.v.) and then sacrificed 90 minutes later (**Figure 2C** and **D**). BM-MSC transfer reduced 3 kDa-Dextran leakage into the brain parenchyma (**Figure 2C** and **D**) and decreased its presence in the peripheral blood (**Figure 2E**). These results illustrate that BM-MSC transfer protects BBB integrity in Tg-SwDI/B mice.

### Bone marrow mesenchymal stem cell transfer mitigates neural inflammation in cerebral amyloid angiopathy

**Figure 3:**
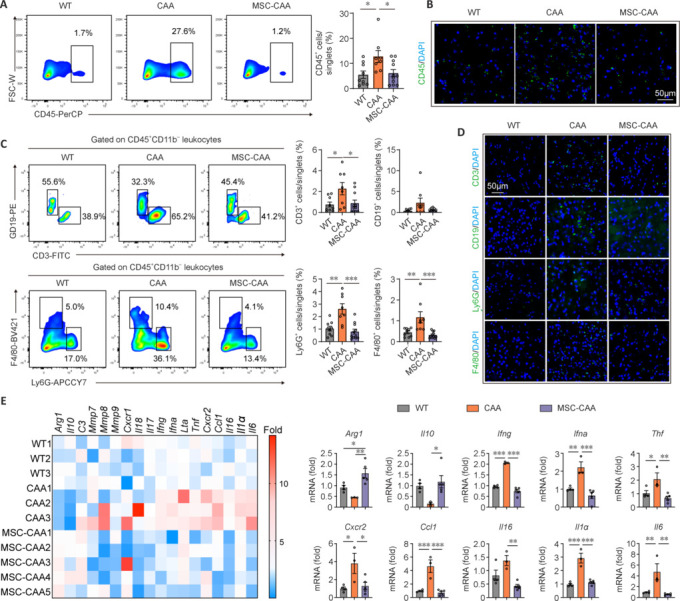
BM-MSC transfer reduces neural inflammation in CAA. Tg-SwDI/B mice with C57/BL6 background received BM-MSCs (2 × 10^6^ cells per mouse, i.v., in two doses with a 3-day interval) starting at 8 months of age. Behavioral tests were conducted starting on day 7, and the mice were euthanized on day 11. Age- and sex-matched WT mice served as controls. (A) Leukocyte (CD45^+^) infiltration in the brains of the above-described mice was analyzed via flow cytometry *in vivo*. *n* = 9 in the WT group; *n* = 8 in the CAA group; *n* = 11 in the MSC-CAA group. (B) Immunostaining of CD45 (green) was performed, and representative confocal images are displayed. *n* = 4 per group. (C) Representative flow plots showing infiltrated T lymphocytes (CD45^+^CD11b^–^CD3^+^CD19^–^), B lymphocytes (CD45^+^CD11b^–^CD3^–^CD19^+^), neutrophils (CD45^+^CD11b^+^F4/80^–^Ly6G^+^), and macrophages (CD45^+^CD11b^+^F4/80^+^Ly6G^–^) in the brains of the above-described mice, and the corresponding statistical analysis results are displayed. *n* = 9 in the WT group; *n* = 8 in the CAA group; *n* = 11 in the MSC-CAA group. (D) Immunostaining of CD3, CD19, Ly6G and F4/80 (green) was performed, and representative confocal images are displayed. *n* = 4 per group. (E) mRNA was extracted from the peripheral hemisphere of the above-described mice and subjected to quantitative reverse transcription-polymerase chain reaction. Left: The expression levels of related mRNAs in each individual were visualized via a heatmap. Right: Statistical analysis was performed to compare the expression levels of related mRNAs among the three groups. *n* = 3 in the WT group, *n* = 3 in the CAA group, and *n* = 5 in the MSC-CAA group. *P < 0.05, **P < 0.01, ***P < 0.001 (one-way analysis of variance followed by Dunnett’s test). BM-MSCs: Bone marrow mesenchymal stem cells; CAA: cerebral amyloid angiopathy; DAPI: 4′,6-diamidino-2-phenylindole; WT: wild-type.

In addition to causing BBB disruption, CAA triggers neuroinflammation, which is characterized by immune cell infiltration and a surge of inflammatory cytokines. This inflammatory response is believed to accelerate the progression of cognitive impairment. To examine neural inflammation in Tg-SwDI/B mice after transferring BM-MSCs, we analyzed immune cell infiltration in the brain parenchyma using flow cytometry and immunostaining (**Figure 3A–D**). We found that the percentages of T cells (CD45^+^CD3^+^CD11b^–^CD19^–^), B cells (CD45^+^CD3^–^CD11b^–^CD19^+^), neutrophils (CD45^+^CD11b^+^F4/80^–^Ly6G^+^), and macrophages (CD45^+^CD11b^+^F4/80^+^Ly6G^–^) were increased in the brain parenchyma of Tg-SwDI/B mice compared with the findings in WT mice. The expression of genes encoding multiple proinflammatory cytokines and chemokines (e.g., *Ifng*, *Ifna*, *Tnf*, *Cxcr2*, *Ccl1*, *Il1a*, and *Il6*) was elevated (**Figure 3E**, *Ifng P* < 0.001 between the WT group and the CAA group; *Ifna P* = 0.0211 between the WT group and the CAA group; *Tnf* *P* = 0.0293 between the WT group and the CAA group; *Cxcr2* *P* = 0.0218 between the WT group and the CAA group; *Ccl1* *P* < 0.001 between the WT group and the CAA group; *Il1a* *P* < 0.001 between the WT group and the CAA group; *Il6 P* = 0.0051 between the WT group and the CAA group), while the gene expression of anti-inflammatory markers (e.g., *Il10*) was decreased in the brain parenchyma of Tg-SwDI/B mice compared with the findings in WT mice (*Il10 P* = 0.0216 between the CAA group and the MSC-CAA group; **Figure 3E**). BM-MSC transfer reduced the brain infiltration of these immune cells in Tg-SwDI/B mice (**Figure 3A–D**), without affecting the leukocyte composition of the peripheral blood (**Additional Figure 1**). Furthermore, BM-MSC treatment reduced the expression of genes encoding proinflammatory molecules and increased the expression of genes encoding anti-inflammatory molecules in CAA lesions (*Ifng P* < 0.001 between the CAA group and the MSC-CAA group; *Ifna P* < 0.001 between the CAA group and the MSC-CAA group; *Tnf P* = 0.0160 between the CAA group and the MSC-CAA group; *Cxcr2 P* = 0.0298 between the CAA group and the MSC-CAA group; *Ccl1 P* < 0.001 between the CAA group and the MSC-CAA group; *Il1a P* < 0.001 between the CAA group and the MSC-CAA group; *Il6 P* = 0.0053 between the CAA group and the MSC-CAA group; **Figure 3E**). These results illustrate that adoptive BM-MSC transfer relieves neuroinflammation in the brains of CAA mice.

### Bone marrow mesenchymal stem cell transfer promotes migrasome production by neutrophils

**Figure 4:**
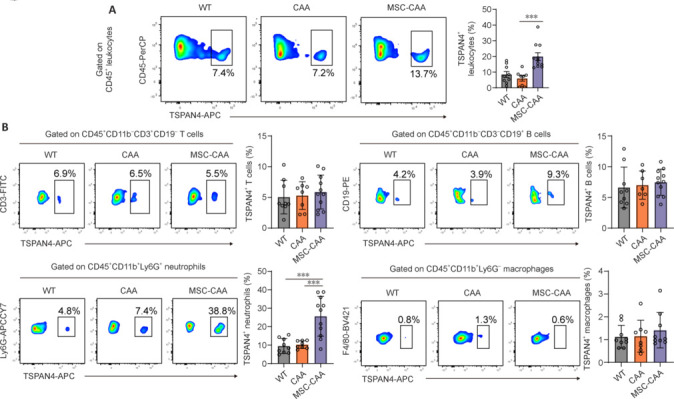
The transfer of BM-MSCs enhances the production of migrasomes from neutrophils. Tg-SwDI/B mice with C57/BL6 background received BM-MSCs (2 × 10^6^ cells per mouse, i.v., in two doses with a 3-day interval) starting at 8 months of age. Behavioral tests were conducted starting on day 7, and the mice were euthanized on day 11. Age- and sex-matched WT mice served as controls. The brain cells from the above-described mice were analyzed via flow cytometry. (A, B) TSPAN4 expression in leukocytes (CD45^+^), T lymphocytes (CD45^+^CD11b^–^CD3^+^CD19^–^), B lymphocytes (CD45^+^CD11b^–^CD3^–^CD19^+^), neutrophils (CD45^+^CD11b^+^F4/80^–^Ly6G^+^), and macrophages (CD45^+^CD11b^+^F4/80^+^Ly6G^–^) was calculated. *n* = 9 in the WT group; *n* = 8 in the CAA group; *n* = 11 in the MSC-CAA group. ***P < 0.001 (one-way analysis of variance followed by Dunnett’s test). BM-MSCs: Bone marrow mesenchymal stem cells; CAA: cerebral amyloid angiopathy; i.v.: intravenously; WT: wild-type.

We previously found that migrasomes produced by immune cells significantly affect outcomes in CAA (Hu et al., 2023). Therefore, we investigated how BM-MSC transfer influences the production of migrasomes by various immune cells in CAA. Flow cytometry and immunostaining showed increased TSPAN4 expression in neutrophils of BM-MSCs treated CAA model brains, while other immune cells like macrophages, T cells, and B cells exhibited stable TSPAN4 levels (**Figure 4A** and **B**). No significant changes in migrasome production by leukocytes in the peripheral blood were observed (**Additional Figure 2**). These findings indicate that BM-MSC transfer enhances migrasome production in intra-lesional neutrophils in CAA mice.

### Bone marrow mesenchymal stem cells promote mitochondrial quality control in neutrophils via migrasomes

**Figure 5:**
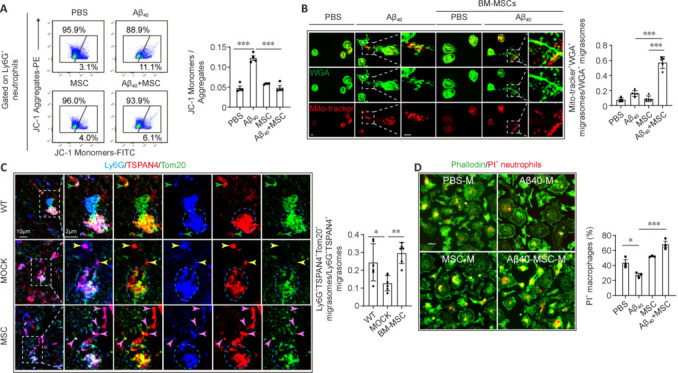
BM-MSCs support migrasome-dependent mitochondrial quality control in neutrophils. (A, B) Bone marrow-derived neutrophils were isolated from wild-type (WT) mice and treated with Aβ_40_ (20 μg/mL for 2 hours), with or without co-culture with BM-MSCs at a ratio of 5:1. (A) The mitochondrial membrane potential of neutrophils (Ly6G^+^) was measured using JC-1 dye and analyzed by flow cytometry. JC-1 monomers emit green fluorescence (**~**529 nm, detected with the FITC channel), while JC-1 aggregates emit red fluorescence (**~**590 nm, detected with the PE channel). Each experiment was performed four times. (B) Migrasome formation and mitocytosis in neutrophils were assessed using Mito-tracker and WGA staining. Scale bar: 2 μm. The ratios of Mito-tracker^+^WGA^+^ migrasomes were calculated. Each experiment was performed five times. (C) Tg-SwDI/B mice on a C57BL/6 background received BM-MSCs (2 × 10^6^ cells per mouse, administered intravenously in two doses with a 3-day interval) starting at 8 months of age. Coronal brain sections from the recipients were collected and subjected to immunostaining for Ly6G (blue), TSPAN4 (red), and Tom20 (green) to evaluate the mitocytosis of neutrophils in the brain. The ratios of Ly6G^+^TSPAN4^+^Tom20^+^ migrasomes were calculated. *n* = 6 in each group. (D) Bone marrow-derived neutrophils were isolated from WT mice, treated with Aβ40 (20 μg/mL for 2 hours), and co-cultured with BM-MSCs (5:1). Neutrophil-derived migrasomes were isolated and applied to primary mouse bone marrow-derived macrophages (50 μg/mL for 24 hours). After treatment, the phagocytic functions of the macrophages were assessed through immunostaining. Scale bar: 20 μm. Each experiment was conducted three times. *P < 0.05, **P < 0.01, ***P < 0.001 (one-way analysis of variance followed by Dunnett’s test). Aβ: Amyloid-beta; BM-MSCs: bone marrow mesenchymal stem cells; PBS: phosphate buffered saline; WGA: wheat germ agglutinin; WT: wild-type.

Previous research has indicated that neutrophils can expel damaged mitochondria by encapsulating them in migrasomes, thereby maintaining mitochondrial quality within the cell (Jiao et al., 2021). We thus hypothesized that the enhanced migrasome production by neutrophils observed after BM-MSC treatment could improve mitochondrial turnover in neutrophils. Aβ_40_ was used to treat neutrophils *in vitro* to simulate the microenvironment of a CAA lesion. We observed marked mitochondrial damage in neutrophils after Aβ_40_ treatment (*P* < 0.001 between the PBS group and the Aβ_40_ group; *P* < 0.001 between the Aβ_40_ group and the Aβ_40_ + MSC group; **Figure 5A**). Subsequently, migrasome-mediated mitocytosis was activated. Co-culture with BM-MSCs significantly enhanced the efficiency of migrasome-mediated mitocytosis in Aβ_40_-treated neutrophils, thus relieving the intracellular burden of damaged mitochondria (*P* < 0.001 between the Aβ_40_ group and the Aβ_40_ + MSC group; *P* < 0.001 between the MSC group and the Aβ_40_ + MSC group; **Figure 5A** and **B**). Consistent with this, immunostaining of mouse brain slices showed that BM-MSC treatment increased the number of mitochondria in migrasomes produced by neutrophils in the brain parenchyma of Tg-SwDI/B mice (*P* = 0.0376 between the WT group and the Mock group; *P* = 0.0033 between the Mock group and the BM-MSC group; **Figure 5C**).

We next explored the impact of neutrophil-derived migrasomes on other immune cells and surrounding neural cells, with an emphasis on scavenger, macrophage lineage cells. We found that neutrophil-derived migrasomes had little impact on cellular proliferation or cell death in organotypic brain slice cells or spleen cells (**Additional Figure 3**). Interestingly, neutrophil-derived migrasomes significantly enhanced the phagocytic function of primary bone marrow–derived macrophages (*P* = 0.0269 between the PBS group and the Aβ_40_ group; *P* < 0.001 between the Aβ_40_ group and the Aβ_40_ + MSC group; **Figure 5D**). These findings illustrate that neutrophils renewed their mitochondria through migrasome-mediated mitocytosis, thereby enhancing their own clearance by macrophage lineage cells.

### Bone marrow mesenchymal stem cells transfer functional mitochondria to neutrophils via migrasomes, aiding the removal of damaged mitochondria from neutrophils

**Figure 6:**
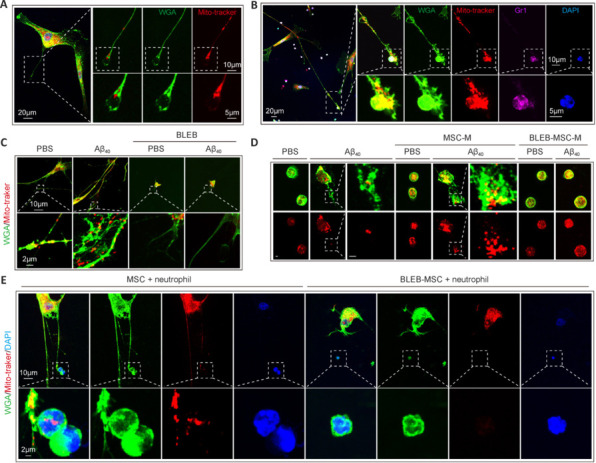
The transfer of BM-MSCs delivers functional mitochondria to neutrophils through migrasomes, facilitating the elimination of damaged mitochondria. (A) BM-MSCs were seeded on fibronectin-coated coverslips for six hours and then subjected to MitoTracker and WGA staining. Each experiment was conducted three times, and representative images are shown. (B) Bone marrow-derived neutrophils were isolated from WT mice and co-cultured with BM-MSCs at a ratio of 5:1 for 2 hours. The transfer of functional mitochondria from BM-MSCs to neutrophils was assessed using MitoTracker (red), WGA (green), Ly6G (purple), and DAPI (blue) staining. Each experiment was conducted three times, and representative images are presented. (C) BM-MSCs were pre-treated with BLEB (50 μM for 24 hours) to inhibit migrasome formation and then treated with Aβ40 (20 μg/mL for 2 hours). Migrasome formation and mitocytosis in BM-MSCs were evaluated via MitoTracker and WGA staining. Each experiment was performed four times. (D) BM-MSCs were pretreated with BLEB (50 μM for 24 hours) to inhibit migrasome formation, and the resulting migrasomes were isolated. Bone marrow-derived neutrophils were isolated from WT mice and treated with Aβ_40_ (20 μg/mL for 2 hours) along with migrasomes from BM-MSCs with or without BLEB pre-treatment. Migrasome formation and mitocytosis in neutrophils were assessed using MitoTracker and WGA staining. Scale bars: 2 μm. Each experiment was performed four times. (E) BM-MSCs were pre-treated with blebbistatin (BLEB, 50 μM for 24 hours) to inhibit migrasome formation. Bone marrow-derived neutrophils were isolated from WT mice and co-cultured with BM-MSCs at a ratio of 5:1 for 2 hours. The transfer of functional mitochondria from BM-MSCs to neutrophils was evaluated using MitoTracker (red), WGA (green), and DAPI (blue) staining. Each experiment was conducted three times, and representative images are shown. Aβ: Amyloid-beta; BLEB: blebbistatin; BM-MSCs: bone marrow mesenchymal stem cells; DAPI: 4′,6-diamidino-2-phenylindole; WGA: wheat germ agglutinin; WT: wild-type.

We previously found that BM-MSCs produce migrasomes under physiological conditions (Li et al., 2023a). Prior studies have shown that BM-MSCs transfer their healthy mitochondria to neurons, thereby restoring mitochondrial function and conferring neuroprotection (Zhao et al., 2021). Thus, we hypothesized that BM-MSCs may donate healthy mitochondria to neutrophils, aiding in the removal of damaged mitochondria and preventing oxidative stress buildup. This could trigger a cycle in which migrasomes boost further migrasome production. To test this hypothesis, we stained BM-MSC-derived migrasomes with WGA and MitoTracker to detect mitochondria (**Figure 6A**). Blocking migrasomes with BLEB reduced mitocytosis (**Figure 6C**).

We next asked whether BM-MSCs transfer mitochondria to neutrophils through migrasomes. BM-MSCs containing mitochondria labeled with MitoTracker were co-cultured with primary neutrophils. After 2 hours, we labeled BM-MSC–derived migrasomes with WGA and neutrophils with Ly6G. Our results demonstrated that BM-MSCs transfer mitochondria to neutrophils via migrasomes (**Figure 6B**). Inhibiting BM-MSC migrasome production using BLEB reduced this mitochondrial transfer (**Figure 6E**). Additionally, Aβ_40_ treatment enhanced the ability of BM-MSCs to release mitochondria through migrasomes (**Figure 6C**), while inhibiting BM-MSC migrasome production abolished this effect (**Figure 6C**). Next, we examined whether BM-MSC–derived migrasomes induced neutrophil mitochondrial renewal. Applying BM-MSC migrasomes to Aβ_40_-stimulated neutrophils increased mitocytosis (**Figure 6D**), while BM-MSC-derived EVs (a negative control for migrasomes) had no such effect (**Figure 6D**). This suggests that BM-MSCs deliver functional mitochondria to neutrophils via migrasomes, promoting the clearance of damaged mitochondria.

## Discussion

The current study demonstrates that BM-MSCs offer protection against CAA. BM-MSC-derived migrasomes, which contain functional mitochondria, help maintain mitochondrial quality in neutrophils by enhancing mitocytosis. Thus, BM-MSCs represent a promising therapeutic approach for CAA.

Given the severe disability and high mortality associated with untreated CAA, effective treatment is crucial. Current clinical management of CAA involves addressing symptoms, for example by monitoring blood pressure and intracranial pressure, avoiding antiplatelet agents and anticoagulants unless necessary, improving cognitive function, and considering surgical intervention for cerebral hemorrhage (Kozberg et al., 2021; Cozza et al., 2023). However, effective treatments for these symptoms are lacking. There is a significant unmet need for novel therapeutic strategies that can halt or slow the progression of CAA.

MSCs show promise for cell-based therapies targeting brain injury and diseases (Shen et al., 2025; Zhang et al., 2025). Research has indicated that MSC transfer can improve cognitive impairment in AD, which shares similarities with CAA in terms of amyloid protein deposition and comorbidity rates (Giovannelli et al., 2023; Cone et al., 2021). However, the therapeutic efficacy of BM-MSCs toward CAA remains unexplored. In our study, we found that BM-MSC treatment improved cognitive function in Tg-SwDI/B mice, as evidenced by the Morris water maze and novel object recognition tests. Additionally, BM-MSC–treated mice showed increased neuronal activation and reduced amyloid protein deposition. A previous study suggested that cognitive dysfunction and amyloid deposition in CAA may result from BBB disruption and subsequent neuroinflammation (Hu et al., 2023). Our findings also indicate that BM-MSC treatment preserved BBB integrity and reduced neuroinflammation in Tg-SwDI/B mice.

Accumulating evidence supports the therapeutic efficacy of BM-MSCs in cerebral vascular disease and cognitive impairment (Cone et al., 2021; Xu et al., 2022; Hu et al., 2025), though the underlying mechanisms are not yet fully understood. Migrasomes, newly identified organelles generated during cellular migration, play a key role in mitocytosis, which maintains mitochondrial membrane potential and respiration by removing damaged mitochondria from migrating cells (Jiao et al., 2021). In our study, we observed activated neutrophil mitocytosis in the parenchyma of Tg-SwDI/B mice following BM-MSC transfer, which enhanced neutrophil clearance by macrophages and alleviated neuroinflammation.

While the exact mechanisms underlying the beneficial effects of MSCs remain unclear, emerging data suggest that MSC-derived EVs may provide similar benefits to MSCs themselves (Giovannelli et al., 2023; Li et al., 2023b). Research indicates that BM-MSC–derived migrasomes, which contain soluble proteins, can aid in recovery from cerebral vascular diseases (Li et al., 2023a). Additionally, MSCs have been shown to rescue dying immune cells by transferring functional mitochondria to them (Zhao et al., 2021). Migrasomes, which are relatively large organelles, can carry functional mitochondria and facilitate intercellular communication (Yu and Yu, 2022). Our study showed that BM-MSCs convey migrasomes containing active mitochondria to neutrophils, thereby increasing neutrophil mitocytosis and ensuring stable mitochondrial function.

This study had some limitations that should be noted. Regarding the clinical relevance of our findings, while cerebral microbleeds and lobar hemorrhages are pathological hallmarks of CAA, animal models predominantly concentrate on amyloid accumulation, which restricted our capacity to assess the impact of BM-MSCs on these hemorrhagic manifestations. However, studies have demonstrated the efficacy of MSC in treating spontaneous intracerebral hemorrhage (Bedini et al., 2018; Yang et al., 2024). Consequently, BM-MSCs are a potential therapeutic strategy for CAA in a clinical context.

In conclusion, BM-MSCs offer protection against CAA by improving cognitive function, reducing neuroinflammation, and preserving BBB integrity. They transfer mitochondria to neutrophils via migrasomes, promoting neutrophil mitocytosis and facilitating macrophage clearance. Further studies are needed to fully explore the potential of BM-MSCs in CAA treatment and to investigate their therapeutic value in patients with CAA.

## Additional files:

***Additional Table 1:*** *The primers used for quantitative reverse transcription-polymerase chain reaction.*

***Additional Figure 1:*** *BM-MSCs transfer has little effect on leukocyte composition in peripheral blood.*

Additional Figure 1 BM-MSCs transfer has little effect on leukocyte composition in peripheral blood.
Tg-SwDI/B mice with C57/BL6 background received BM-MSCs (2 × 10^6^ cells per mouse, i.v., in two doses with a 3-day interval) starting at 8 months of age. Behavioral tests were conducted starting on day 7, and mice were euthanized on day 11. Age- and sex-matched wild type (WT) mice were served as controls. Peripheral blood of above mice was isolated and subjected to flow cytometry. Representative flow plots showing T lymphocytes (CD45^+^CD11b^-^CD3^+^CD19^-^), B lymphocytes (CD45^+^CD11b^-^CD3^-^CD19^+^) (A), neutrophils (CD45^+^CD11b^+-^Ly6G^+^), and macrophages (CD45^+^CD11b^+^Ly6G^-^) (B) in the peripertal blood of above mice, and the corresponding statistical analysis were displayed. *n* = 4 in each group. BM-MSCs: bone marrow mesenchymal stem cells; CAA: cerebral amyloid angiopathy; WT: wild-type.

***Additional Figure 2:*** *BM-MSCs transfer has little effect on migrasome production in peripheral leukocytes.*

Additional Figure 2 BM-MSCs transfer has little effect on migrasome production in peripheral leukocytes.
Tg-SwDI/B mice with C57/BL6 background received BM-MSCs (2 × 10^6^ cells per mouse, i.v., in two doses with a 3-day interval) starting at 8 months of age. Behavioral tests were conducted starting on day 7, and mice were euthanized on day 11. Age- and sex-matched wild type (WT) mice served as controls. Peripheral blood of above mice was isolated and subjected to flow cytometry .Representative flow plots showing Leukocyte (CD45^+^) (A), T lymphocytes (CD45^+^CD11b^-^CD3^+^CD19^-^) (B), B lymphocytes (CD45^+^CD11b^-^CD3^-^CD19^+^) (C), neutrophils (CD45^+^CD11b^+^F4/80^-^Ly6G^+^) (D), and macrophages (CD45^+^CD11b^+^F4/80^+^Ly6G^-^) (E) in the peripertal blood of above mice, and the corresponding statistical analysis were displayed. *n* = 4 in each group. BM-MSCs: bone marrow mesenchymal stem cells; CAA: cerebral amyloid angiopathy; WT: wild-type.

***Additional Figure 3:*** *Neutrophil-derived migrasomes have minimal effects on cell proliferation and death.*

Additional Figure 3 Neutrophil-derived migrasomes have minimal effects on cell proliferation and death.
Bone marrow-derived neutrophils were isolated from WT mice, treated with Aβ_40_ (20 μg/mL for 2 hours), and co-cultured with BM-MSCs at a ratio of 5:1. Neutrophil-derived migrasomes were then isolated and applied to spleen cells (**A, B**) and organotypic brain slices (**C, D**) at a concentration of 50 μg/mL for 24 hours. (**A** and **C**) Dead cells were identified using a Live/Dead fixable stain kit. Each experiment was repeated four times. (**B** and **D**) Protein expression of Ki67 was analyzed by flow cytometry, with no significant differences found across the four experiments. Aβ: Amyloid-β; BM-MSCs: bone marrow mesenchymal stem cells; WT: wild-type.

## Data Availability

*All relevant data are within the paper and its Additional files*.
