# Supplementary figures and images for: Bone marrow mesenchymal stem cells protect against cerebral amyloid angiopathy by enhancing neutrophil mitocytosis
Source: Neural Regen Res. 2025 Jun 19;21(7):3178–86. doi: 10.4103/NRR.NRR-D-24-01273 (PMC13378942; doi:10.4103/NRR.NRR-D-24-01273)

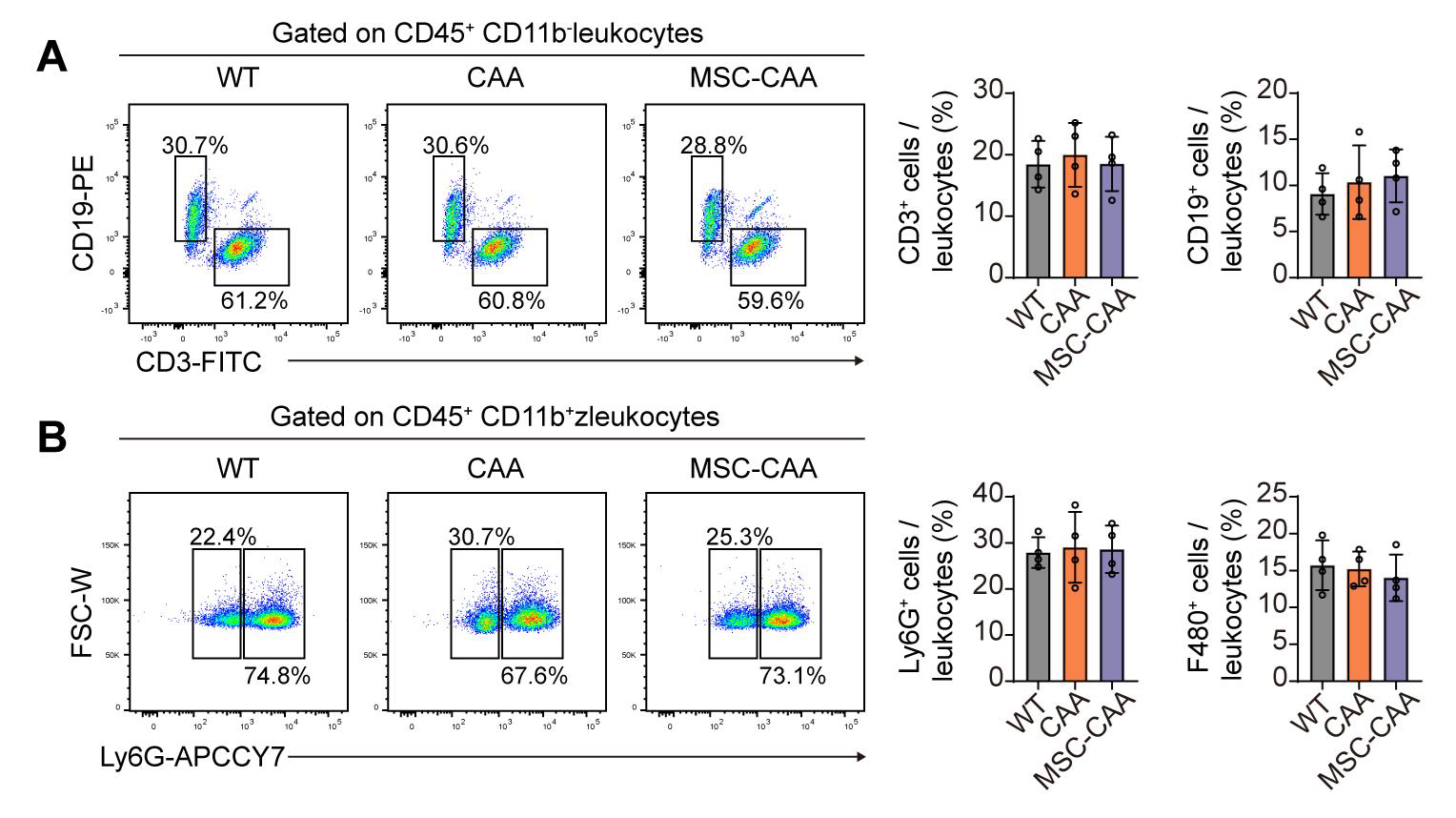

Supplement: Supplementary file 1 [file NRR-21-3178_Suppl1.tif]

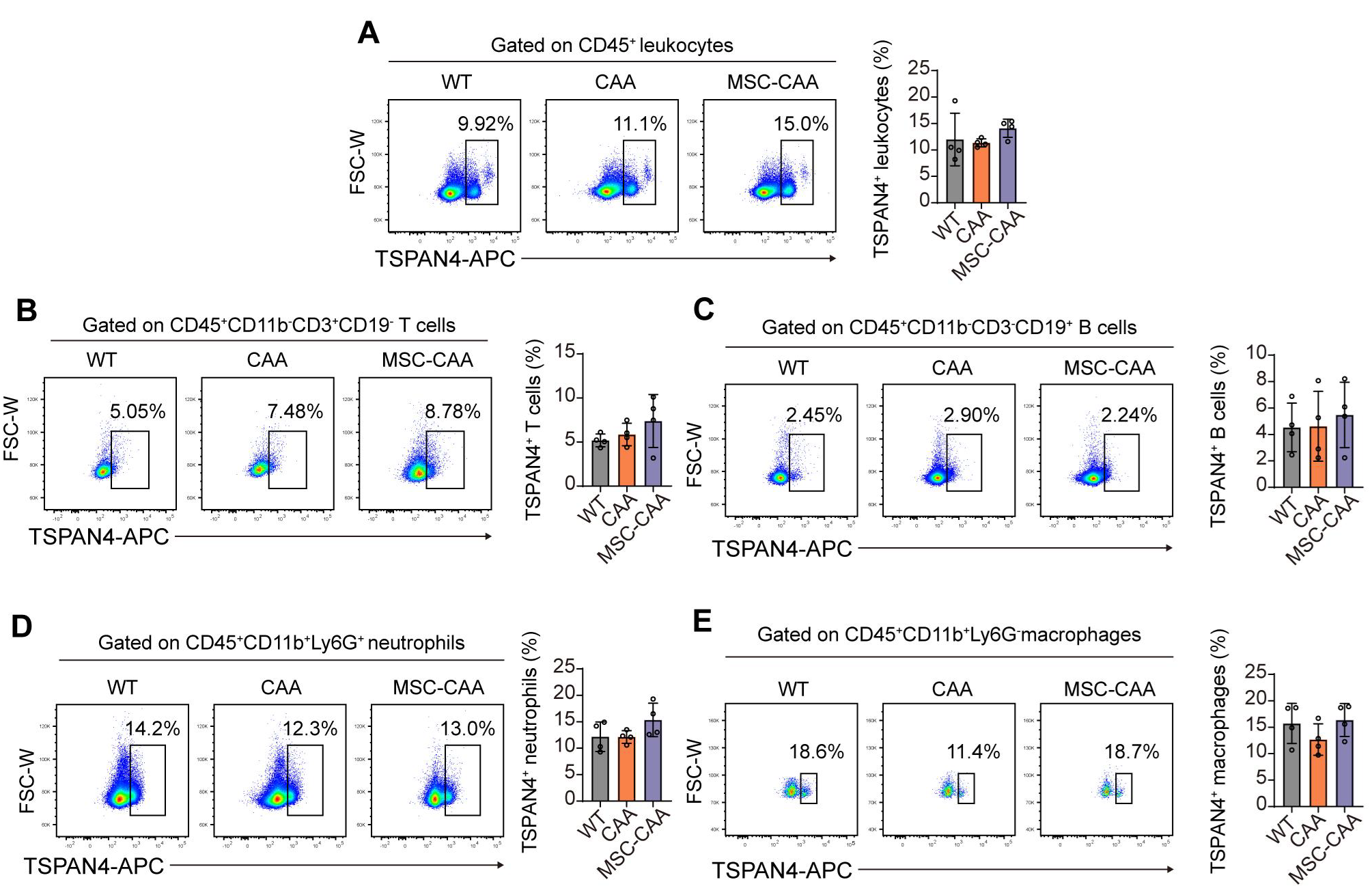

Supplement: Supplementary file 2 [file NRR-21-3178_Suppl2.tif]

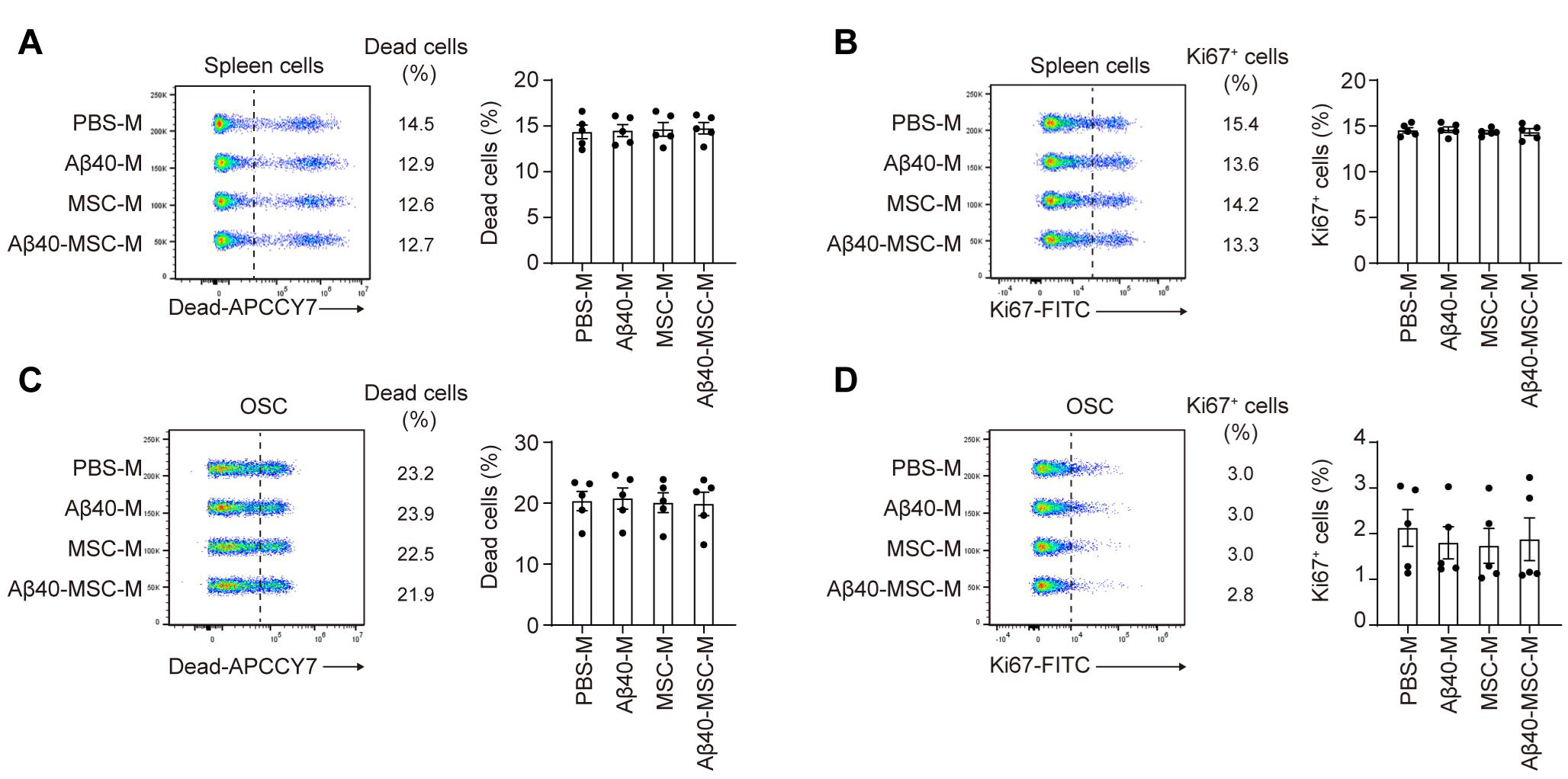

Supplement: Supplementary file 3 [file NRR-21-3178_Suppl3.tif]
